# Valid and Invalid Indications for Osteopathic Interventions: A Systematic Review of Evidence-Based Practices and French Healthcare Society Recommendations

**DOI:** 10.7759/cureus.49674

**Published:** 2023-11-29

**Authors:** Zeinab M Khalaf, Pierre Margulies, Mohamad K Moussa, Yoann Bohu, Nicolas Lefevre, Alexandre Hardy

**Affiliations:** 1 Endocrinology, Diabetes, and Metabolism, clinique du Sport, Paris, FRA; 2 Osteopathic Medicine, Cabinet d'Expertise, Paris, FRA; 3 Orthopedic Surgery, Clinique du Sport, Paris, FRA; 4 Orthopedic Surgery, Clinique du Dport, Paris, FRA

**Keywords:** doctor of osteopathy, osteopathy treatement, manipulative therapy, manual therapy, osteopathy indication

## Abstract

The main aim of this study was to determine the level of evidence in the literature for the main indications of osteopathy as recommended by the French osteopathy societies.

This systematic review followed the 2020 Preferred Reporting Items for Systematic Reviews and Meta-Analyses (PRISMA) guidelines and evaluated articles published between January 2012 and January 2022 with one modification: when level one evidence studies were available, level two to five studies were excluded. Sources included PubMed, the Cochrane library, the French National Health Authority (HAS) and its affiliates. Inclusion criteria were level one published studies on the indications for osteopathic treatment in French and English, and level two to three studies when no level one studies were available. The level of evidence assessment was based on the Oxford Centre for Evidence-Based Medicine (OCEBM) Levels of Evidence classification. The primary outcome was the level of evidence in the literature supporting osteopathic practices. The secondary outcome was to assess French professional osteopathy recommendations and French HAS guidelines in relation to the scientific literature.

A total of 51 articles and nine recommendations from the HAS and its affiliates met the inclusion criteria for the systematic review. Analysis of the studies revealed 41 osteopathic indications from French osteopathy societies for musculoskeletal, neurosensory, psychological, pediatric, gynecological, digestive, and pulmonary disorders. High-level scientific evidence supported the use of osteopathy for low back pain, sciatica, cervical radiculopathy, and ankle sprain. There was moderate evidence for tension headache, temporomandibular joint disorder, endometriosis, and low back and pelvic pain in pregnant women. HAS recommended five indications, while nine indications were supported in the scientific literature.

Osteopathy has been shown to have evidence-based benefits for a range of conditions, in particular for musculoskeletal and neurosensory disorders.

## Introduction and background

Osteopathy is a complementary medical practice involving manual manipulation to treat the joints and myofascial system function [1-10]. It was first described in the United States in the 19th century and has been developed and is practiced worldwide [2, 11, 12]. In France, the Kouchner law of March 2002 created the title "osteopath" for these non-healthcare professionals (non-doctors, non-physiotherapists) [6]. The decree of March 25, 2007, regulated its practice, making it possible to consult these professional therapists for first-line treatment [13, 14]. It is important to note, however, that this specialty includes several backgrounds of non-healthcare professionals as well as healthcare professionals such as doctors and physical therapists. The law states that osteopaths may perform external, non-instrumental manipulations for the prevention and treatment of functional disorders but not for organic diseases requiring medical, surgical, or drug treatments or physical agents [13, 14]. Osteopaths are represented by several societies in France depending on the practitioner's background [15].

The reasons for consulting osteopaths are diverse, but musculoskeletal pain is the main cause, representing 61% of visits. Other reasons include perinatal and pediatric conditions (11.8%), cranial and neurological disorders (9.1%), visceral disorders (5%), and preventive care (0.2%) [2, 16]. Osteopathic consultations for children are often for sleep disorders, unexplained crying, cranial asymmetries, and gastroesophageal reflux [2, 16].

Osteopathy includes numerous techniques, for example, osteopathic manipulative treatment (OMT), which uses hands-on interventions for diagnosis and treatment, and myofascial release (MFR) for targeting fascial tension [4-10]. Additional methods such as cervical, visceral, and thoracic spine manipulation focus on specific regions and joints [5, 7, 17-19] in particular, balanced ligamentous tension, diaphragm doming, indirect diaphragm release, high-velocity low amplitude, lymphatic pump, and rib raising techniques [2]. Pediatric integrative manual therapy (PIMT) has been developed for infants and children [20].

Despite the marked increase in the number of scientific articles published in recent decades, osteopathy is still considered to be an "unconventional healthcare practice" whose benefits and risks have not been sufficiently defined.

The aim of this study was to perform a systematic review (SR) of publications in the literature with a high level of evidence [21] for the main indications of osteopathy. The secondary aim was to assess French osteopathy society recommendations in relation to the existing literature and guidelines from the French National Health Authority (HAS), which is the highest healthcare regulatory body in France.

## Review

Material and methods

This systematic review was performed according to the 2020 Preferred Reporting Items for Systematic Reviews and Meta-Analyses (PRISMA) guidelines [22], with one modification: when level one evidence studies were available, level two to five studies were excluded.

Eligibility Criteria

Inclusion criteria were accessible, level one evidence, French and English language studies that evaluated the indications for osteopathy, such as systematic reviews, meta-analyses, and randomized clinical trials (RCT), and level two to three evidence studies, when no level one studies were available. Any official recommendations available on the sites of French professional healthcare organizations and societies were included.

The level of evidence assessment was based on the Oxford Centre for Evidence-Based Medicine (OCEBM) Levels of Evidence classification [21].

Exclusion criteria were level two to five evidence studies when a level one study was available, unavailable abstracts, non-retrievable manuscripts, manual treatments that were not osteopathic, and articles published before January 2012.

Outcomes

The primary outcome was the level of evidence in the literature supporting osteopathic practices [21].

The secondary outcome was to assess French professional osteopathy recommendations and French HAS guidelines in relation to the scientific literature.

Search Strategy

An in-depth search of the literature from January 2012 to January 2022 was performed in the PubMed and Cochrane databases. Keywords included "specific indications" AND ("osteopathic manipulative therapy," OR "manual therapy", OR "osteopathic manipulative treatment", OR "osteopathic manipulative medicine"). The search also included French HAS (previously called ANAES for Agence nationale d'accréditation et d'évaluation en santé) recommendations using keywords for "specific indications" AND ("ostéopathie" OR "thérapie manuelle" OR "manipulation", OR "recommendation de bonne pratique"). Indication keywords were based on the indications recommended by French osteopathy organizations, syndicates, and societies.

Study Selection

Two independent researchers conducted the research and independently assessed the articles for inclusion or exclusion based on predetermined criteria. In cases where there was a discrepancy or uncertainty regarding the inclusion or exclusion of an article, a third researcher was consulted to provide additional input and help reach a consensus decision. Duplicates were removed by an automatic tool using Rayan Software® [23]. A three-step process was followed for PubMed and Cochrane articles. First, the title was screened for inclusion based on eligibility criteria and to exclude duplicates. Then, the abstract was analyzed to confirm inclusion and to group articles by indication, and categories such as preventive treatment, musculoskeletal complaints, neurosensory complaints, digestive complaints, pulmonary complaints, pediatric issues, and women's health concerns were created. Finally, the full-text manuscript was analyzed for data extraction.

All recommendations from French healthcare or regulatory organizations and societies for osteopathic practices corresponding to the specific indication were included.

Data Extraction

Data extraction included the main indication for each disease category, manual techniques used, conclusion, recommendations, and level of evidence.

Data Synthesis

In the review, a narrative synthesis was performed to provide a comprehensive summary and interpretation of the findings from included studies. These studies were grouped together into predefined categories, such as preventive treatment, musculoskeletal disorders, neurosensory disorders, digestive disorders, pulmonary disorders, pediatric disorders, and women's health. A meta-analysis or a quantitative summary could not be performed because of the type of analysis.

Results

Study Selection

**Figure 1:**
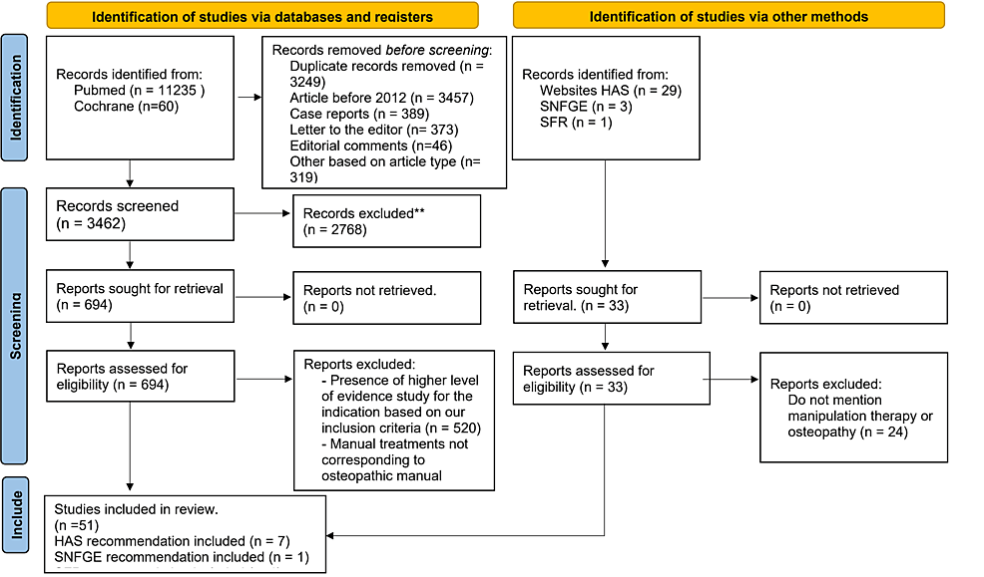
PRISMA 2020 flow diagram for the systematic review PRISMA - Preferred Reporting Items for Systematic Reviews and Meta-Analyses, HAS- Haute autorité de santé (French National Authority for Health), SFR- Société Francaise de Rhumatologie (French Society of Rheumatology), SNFGE- Société savante médicale française d’hépato-gastroentérologie et d’oncologie digestive (French National Society of Gastroenterology and Digesstive Oncology)

A total of 11,295 articles were initially identified through database searches. After removing duplicates (3249), articles before 2012 (3457), and other low-evidence studies such as case reports, editorial comments, letters, and opinions, 3462 articles remained for title and abstract screening. Following the screening process, 694 articles were considered to be potentially eligible for inclusion, and their full-text versions were assessed for eligibility. A total of 51 articles met the inclusion criteria and were included in the SR. Eight HAS guidelines were also included in the analysis (Figure 1).

Indications for Osteopathy

Based on the analysis of French osteopathy organization and society guidelines, 41 osteopathic indications were evaluated (Appendix 1, Table 1) [24-29].

**Table 1:** Summary of osteopathy indications GER - gastroesophageal reflux, ADHD - attention deficit hyperactivity disorder

Musculoskeletal	Neurosensory	Pediatric	Gynecological	Digestive	Pulmonary
Low back pain, neck pain, disc herniation and sciatica, cervical radiculopathy, occipital radiculopathy, intercostal neuralgia, ankle sprains, knee sprains, wrist sprain, carpal tunnel syndrome, lateral epicondylitis, medial epicondylitis, temporomandibular joint disorders	Headaches, migraines, vertigo, adult ADHD, sleep disorders – insomnia, professional burnout	Plagiocephaly, torticollis, GER in children, infant colic, low back pain, childhood ADHD, functional abdominal pain, premature infants, breastfeeding facilitation	Fertility disorders, endometriosis, low back pain and pelvic pain during pregnancy, breech presentation, nausea and vomiting during pregnancy	Gastroesophageal reflux, transit disorders, colitis, irritable bowel syndrome	Asthma

Musculoskeletal Indications

Lower back pain (LBP): In one randomized controlled study, Nguyen et al. compared two osteopathic techniques for the treatment of LBP, and found that standard OMT was more beneficial for LBP-specific activity limitations than a control sham treatment, while both techniques were beneficial to functional status [30]. Several systematic reviews showed that OMT reduced LBP and improved function [1, 10, 31]. MFR was shown to be the most effective technique for reducing chronic LBP pain [31].

Neck pain: A systematic review by Gross et al. showed that the effect of cervical manipulation was similar to that of cervical mobilization for pain, function, and patient satisfaction in adults with neck pain [32]. Corp et al. performed a systematic review and concluded that osteopathy, as part of manual therapy, can improve neck pain [33]. A study by Bagagiolo et al. suggested that OMT was effective for musculoskeletal disorders, including neck pain [1]. Masaracchio et al. performed an SR and a meta-analysis and concluded that thoracic spine manipulation (TSM) provided a short-term benefit for neck pain, but further research was needed to determine its long-term effectiveness [18]. An SR and meta-analysis by Fredin et al. concluded that a combination of osteopathy and exercise therapy was not more effective in reducing the severity of neck pain, neck disability, or in improving the quality of life than exercise therapy alone [4].

Idiopathic scoliosis (IS): Czaprowski performed an SR to investigate the efficacy of nonspecific manual therapy (NMT), in particular manual therapy, chiropractic, and osteopathy, in the treatment of children and adolescents with IS [5]. Six studies were included and the results of the small number of studies in the literature were contradictory. These studies had certain methodological biases, such as small patient groups, incomplete descriptions of study groups, lack of follow-up, and an absence of control groups. The author concluded that the efficacy of NMT was limited for this indication [5].

Sciatica and cruralgia: Lewis et al. performed an SR and meta-analysis (MA) to investigate the clinical effectiveness of different treatment strategies for sciatica [34]. They identified 122 relevant studies, including 90 RCTs, and grouped interventions into 21 treatment strategies. The study found that there was a statistically significant improvement in overall recovery following disc surgery, epidural injections, non-opioid analgesia, manipulation (spinal OMT), and acupuncture compared to inactive controls or conventional care [34].

Cervical radiculopathy: Boyles et al. performed an SR of RCTs to investigate the effectiveness of manual therapy in treating cervical radiculopathy [19]. They identified four studies with a high level of evidence and showed that using manual therapy techniques in association with therapeutic exercise is effective in increasing function and range of motion as well as in decreasing pain and disability [19].

Ankle sprain: A RCT by Eisenhart et al. found that patients with acute grade 1-2 ankle sprain who received OMT in the emergency department experienced statistically significant improvement in edema, pain, and range of motion [35]. An SR by Seah et al. showed that the outcome of functional treatment options, including OMT, were statistically better than immobilization for mild-to-moderate ankle sprains in primary care, while severe sprains required a short period of immobilization [36].

Lateral epicondylitis: The results in the literature are not conclusive. An SR by Hoogvliet et al. showed that OMT provided a short-term benefit [37]. Bisset et al. found that local elbow manipulation has an immediate benefit for pain-free grip strength and the pressure pain threshold, but studies were based on a single treatment session and lacked long-term follow-up [38].

Temporomandibular joint disorder (TMD): In an RCT by Cuccia et al., patients who received OMT required less pain medication than those who received standard conservative therapy [6]. This beneficial effect seems to be optimized when combined with orofacial treatment [7]. An SR by Calixtre et al. provided evidence to support the benefits of OMT depending on the technique. They concluded that osteopathy is beneficial but recommended further studies for stronger clinical proof [8]. A meta-analysis by Martins et al. reported a significant improvement in active mouth opening and pain, in particular, a short-term benefit [9].

Armijo-Olivo et al. performed an SR to evaluate the effectiveness of manual therapy and therapeutic exercise for TMD [39]. They found low-level evidence with no clear results to show that exercise was more effective than other conservative treatments for TMD. Nevertheless, the effects of manual therapy alone or in combination with exercises were found to be promising [39].

Neurosensory and Psychological Indications

Tension headache (TH): Krøll et al. performed an SR and MA to investigate the effects of various treatments, including OMT, on the frequency of TH and quality of life. Results showed that OMT could reduce the frequency of headaches and improve the quality of life during follow-up, although the overall level of evidence was low [40]. In a related RCT, Espí-López et al. compared the effect of different OMT techniques on the quality of life of patients with tension-type headaches [41]. They found that all three treatments (suboccipital inhibitory pressure, suboccipital spinal manipulation, and a combination of the two) were effective in improving various dimensions of the patients' quality of life, with the best results with combined treatment. Treatment was found to be effective after as early as four weeks [41].

Migraine headache: Rist et al. performed an SR and MA to investigate the impact of spinal manipulation on migraine pain and disability [42]. An analysis of RCTs with a pooled total of 677 patients showed that spinal manipulation reduced the severity of pain and the duration of migraines. However, these results should be considered preliminary because of the limitations of the included studies [42].

Vertigo: Tramontano et al. performed an SR to investigate the effects of OMT in the management of patients with vertigo and balance disorders. Their results showed a weak benefit to balance disorders across different outcomes in all of the included studies (unclear) [43].

Pediatric Indications

Cranial malformation and plagiocephaly: An RCT by Pastor-Pons et al. showed significantly greater improvement in the average Oblique Diameter (ODDI) for infants in the PIMT group, which decreased from 107.9% to 103.9% than in the control group. Other studies have confirmed these results and suggest that PIMT can improve cranial asymmetries in infants with nonsynostotic occipital plagiocephaly [44, 45].

Torticollis: Pastor-Pons et al. found PIMT to be effective in the treatment of cervical movement limitations in infants with positional plagiocephaly (PP), in particular with significant improvement in active rotation range of motion and neuromotor development. The results of the SR by Ellwood et al. were similar [46]. OMT was found to be moderately effective in increasing the range of motion in patients with torticollis.

Premature births: Lanaro et al. analyzed five RCTs including 1306 infants. They found that osteopathy reduced the length of hospital stay and costs with no reported adverse events [47].

Other pediatric indications: There was insufficient evidence to confirm the effectiveness of manipulative therapies for idiopathic scoliosis [48-50] and ADHD [51]. The results for infant colic were mixed, and methodological limitations made it impossible to draw definite conclusions [52-54]. The study by Jones et al. did not show any significant benefits to OMT for child asthma [55]. A study by Danielo Jouhier et al. showed that OMT did not improve exclusive breastfeeding at one month [56].

Gynecological

Fertility: The SR by Ruffini et al. examined the effectiveness of OMT for infertility. The evaluated outcomes were hormone levels, conception, pregnancy rates, and quality of life [57]. Results suggested that OMT improved conception rates in women with secondary idiopathic infertility. However, the definitions of successful delivery rates and pregnancy complications were inconsistent, and adverse events were not reported [57].

Endometriosis: A prospective cohort study by Daraï et al. showed that OMT improved symptoms and quality of life in patients with colorectal endometriosis [58]. This same group also performed a prospective pilot study evaluating OMT in patients with deep infiltrating endometriosis (DIE) and colorectal involvement that showed significant improvement in quality of life [59].

Back and pelvic pain in pregnancy: Franke et al. found in their systematic review that osteopathy benefits pregnant or postpartum women with LBP [60]. Licciardone et al., in an RCT of 144 pregnant women, determined that osteopathy mitigates back-specific functioning decline in the third trimester [61]. Hall et al. concluded from their review of 1198 pregnant women that evidence is limited for manual therapies addressing pregnancy-related LBP and pelvic pain [62].

Digestive

Gastroesophageal reflux (GERD): Lynen et al. performed an RCT in 70 patients with GERD and found that visceral OMT resulted in a statistically significant improvement in the Quality of Life in Reflux and Dyspepsia questionnaire [17]. Similar results were reported by Eguaras et al. [63].

Constipation: A pilot study by Belvaux et al. in 21 female patients with chronic constipation suggests that visceral OMT could improve functional constipation and defecation disorders [64].

Irritable bowel syndrome (IBS): Attali et al. performed an RCT in 31 consecutive refractory IBS patients and found that visceral osteopathy improved short-term and long-term outcomes in patients with IBS [65].

Pulmonary

Asthma: An SR of manual therapies for the treatment of patients with bronchial asthma by Hondras et al. concluded that there was insufficient evidence to recommend the use of manual therapy for patients with asthma [66].

HAS Recommendation

The indications for osteopathy recommended by the HAS [67-74] are summarized in Table 2.

**Table 2:** Summary of the French National Authority of Healthy recommendation mentioning osteopathy HAS- Haute autorité de santé (French National Authority for Health), SFR- Société Francaise de Rhumatologie (French Society of Rheumatology), SNFGE- Société savante médicale française d’hépato-gastroentérologie et d’oncologie digestive (French National Society of Gastroenterology and Digesstive Oncology), IBS - irritable bowel syndrome

Pathology	Osteopathic Recommendation	Year
Back pain [67]	Recommended, complementary therapy	2019
Neck pain [68]	Recommended (acute)	2003
Sciatica [74]	HAS: No opinion; SFR: Recommended (acute)	2019
Headache [69]	Recommended, complementary therapy (chronic daily headaches)	2004
IBS [72]	HAS: No opinion; SNFGE: Non-conclusive	2016
Cranial deformation [70]	Inconclusive, Not indicated as first-line treatment; possible second-line therapy	2020
Torticollis [70]	Not recommended	
Endometriosis [71]	Recommended	2017

The summary of the included studies and their findings is presented in Table 3.

**Table 3:** Summary of included articles in the systematic review LBP - low back pain, OMT - osteopathic manual therapy, RCT - randomized controlled trial, CLBP - chronic low back pain, MFR - myofascial release, SRs/MAs - systematic reviews/meta-analyses, NSLBP - nonspecific low back pain, CNSNP - chronic nonspecific neck pain, CNCP - chronic non-cancer pain, IBS - irritable bowel syndrome, CPGs - clinical practice guidelines, MT - manual therapy, ET - exercise therapy, IS - idiopathic scoliosis, CR - cervical radiculopathy, TMD - temporomandibular joint disorder, TTH - tension-type headache, GERD - gastroesophageal reflux disease, KESS - Knowles-Eccersley-Scott Symptom Questionnaire, VAS - Visual Analog Scale, FEV1 - forced expiratory volume in one second, FVC - forced vital capacity, AROM - active rotation range of motion, PP- positional plagiocephaly, ODDI - Oblique Diameter Difference Index, PIMT- pediatric integrative manual therapy, NSOP - nonsynostotic occipital plagiocephaly, EDI - Ear Deviation Index, CPI - Cranial Proportional Index, CVA - cranial vault asymmetry, SBA - skull base asymmetry, TCVA - total cranial vault asymmetry, AIMS - Alberta Infant Motor Scale, CMT - congenital muscular torticollis, WHOQOL-BREF - World Health Organization Quality of Life-Brief version, ADHD - attention deficit hyperactivity disorder, QOL - quality of life.

Indication	Year	Study type	Conditions studied	Sample size	Outcome measures	Main findings (recommended or not)	Level of evidence: non-systematic reviews/ highest in systematic rviews
LBP	Franke et al., 2014 [10]	Systematic review of RCT	Nonspecific LBP	307 studies 15 included	Pain relief and functional status	OMT recommended for acute and chronic nonspecific LBP, pregnant, and postpartum women	1
	Nguyen et al., 2021 [30]	RCT	Nonspecific subacute/chronic LBP	394 participants	LBP-specific activity limitations, pain reduction, health-related quality of life, sick leaves, LBP episodes, consumption of analgesics and nonsteroidal anti-inflammatory drugs	OMT has some benefit, but clinical relevance is questionable	1
	Dal Farra et al., 2021 [31]	Meta-analysis	CLBP	10 articles	Pain and functional status	OMT and MFR recommended for CLBP, with MFR having better evidence for pain reduction	1
	Bagagiolo et al., 2022 [1]	Overview of SRs/MAs	Acute/chronic NSLBP, CNSNP, CNCP, pediatric conditions, primary headache, IBS	9 SRs/MAs	Pain, functional status, and safety of OMT for various conditions	OMT recommended for musculoskeletal disorders, limited and inconclusive evidence for others	1
Neck pain	Gross et al., 2010 [32]	Systematic review	Neck pain, with or without cervicogenic headache or radicular findings	27 trials	Pain, function/disability, patient satisfaction, quality of life, and global perceived effect	Osteopathic treatment beneficial, but optimal technique and dose need to be determined	3
	Corp et al., 2021 [33]	Systematic review	Neck and LBP	17 CPGs	Recommended treatment options for use across Europe	Osteopathy beneficial for neck pain treatment, broad consensus for non-pharmacological treatments in Europe	3
	Fredin et al., 2017 [4]	Meta-analysis	Grade I-II neck pain	7 trials	Pain intensity at rest, neck disability, and quality of life	Osteopathy, as part of MT, not more beneficial when combined with ET compared to ET alone	1
	Bagagiolo et al., 2022 [1]	Overview of SRs/MAs	Neck pain	9 SRs/MAs	Efficacy and safety of OMT for various conditions	Osteopathy for neck pain beneficial, promising evidence for musculoskeletal disorders, further research needed	1
Scoliosis	Czaprowski, 2016 [5]	Systematic review	Idiopathic scoliosis (IS) in children and adolescents	6 studies	Efficacy of non-specific manual therapy	Efficacy of non-specific manual therapy for IS cannot be reliably evaluated, further research needed	1
Sciatica and cruralgia	Lewis et al., 2015 [34]	Network meta-analysis	Sciatica and cruralgia	122 studies	Effectiveness of different treatment strategies	Osteopathy (spinal manipulation) beneficial for sciatica, further research needed to determine its effectiveness in a sequential treatment	3
Cervical radiculopathy	Boyles et al., 2011 [19]	Systematic review	CR	4 studies	Effectiveness of manual therapy	Osteopathy beneficial for cervical radiculopathy, high-quality RCTs with control groups needed for clear and effective protocols	1
Ankle sprain	Seah et al., 2017 [36]	Systematic review	Ankle sprain	33 articles	Best practices for managing ankle sprains	Osteopathic manipulation beneficial for mild-to-moderate ankle sprains, short immobilization for severe sprains	3
	Eisenhart et al. 2003 [35]	Randomized controlled trial	Acute ankle injuries	55 patients	Efficacy of OMT in acute ankle injuries	Osteopathy (single OMT session) beneficial for managing acute ankle injuries	1
Medial and lateral epicondylitits	Hoogvliet et al., 2013 [37]	Systematic review	Lateral and medial epicondylitis	12 RCTs	Effectiveness of exercise therapy and mobilization techniques	Moderate evidence for short-term effectiveness, more research needed	1
	Bisset et al., 2005 [38]	Systematic review and meta-analysis	Lateral epicondylalgia (tennis elbow)	28 RCTs	Effectiveness of physical interventions	Limited evidence for effectiveness of local elbow manipulation, orthotics, and taping; more research needed for definitive conclusions	1
TMD	Martins et al., 2016 [9]	Systematic review with meta-analysis	TMD	375 (8 RCTs)	Active mouth opening and pain during active mouth opening	Osteopathy is beneficial for treating TMD, with a larger effect in the short term compared to other conservative treatments	1
	Armijo-Olivo et al., 2016 [39]	Systematic review	TMD	48 RCTs	Effectiveness of MT and therapeutic exercise interventions	No high-quality evidence and thus, there is great uncertainty about the effectiveness of exercise and manual therapy for TMD	1
	Calixtre et al., 2015 [8]	Systematic review	TMD	8 studies	Maximum mouth opening and pain	Osteopathy is beneficial, but further studies with standardized evaluations and better study designs are recommended	1
	Von Piekartz et al., 2013 [7]	Randomized controlled trial	TMD and cervicogenic headache	43	Cervical movement impairment	Osteopathy is beneficial, and manual therapists should look for features of TMD when examining patients with headache	1
	Cuccia et al., 2010 [6]	Randomized controlled trial	TMD	25	Effectiveness of OMT vs conventional conservative therapy	Osteopathy is beneficial, and OMT is a valid option for the treatment of TMD	1
Headaches	Krøll et al., 2021 [40]	Systematic review and meta-analysis	TTH	13 RCTs	TTH frequency, quality of life, pain intensity, stress symptoms	Osteopathy may be beneficial (manual joint mobilization techniques)	1
	Espí-López et al., 2016 [41]	Factorial, randomized, single-blinded, controlled clinical trial	TTH	76	Quality of life (Sort Form-12 questionnaire)	Osteopathy is beneficial for improving some aspects of quality of life	1
	Rist et al., 2019 [42]	Systematic review and meta-analysis	Migraine	677 (pooled)	Migraine days, migraine pain/intensity	Osteopathy may be beneficial for reducing migraine pain and disability	1
Vertigo	Tramontano et al., 2021 [43]	Systematic review	Vertigo and balance disorders	114	Balance function, adverse events	Osteopathy demonstrates weak positive effects on balance function	1
GERD	Lynen et al., 2022 [17]	Randomized controlled trial	GERD	70	Gastrointestinal symptoms, quality of life, medication use, Reflux Disease Questionnaire, Quality of Life in Reflux and Dyspepsia score	Osteopathy may benefit GERD patients, but primary outcome inconclusive	3
	Eguaras et al., 2019 [63]	Randomized, double-blind, placebo-controlled trial	GERD	60	GERD symptoms, Gastroesophageal Reflux Disease Questionnaire, cervical mobility, pressure pain threshold	Osteopathy is useful for improving GERD symptoms, cervical mobility, and pressure pain threshold	2
Constipation	Belvaux et al., 2017 [64]	Pilot study	Chronic constipation in women	21	KESS, stool frequency, Bristol Stool Form scale, oro-anal transit time, bloating, abdominal pain, quality of life, drug use, VAS constipation	Osteopathy has potential benefits for treating functional constipation in women	2
IBS	Attali et al., 2013 [65]	Randomized, cross-over placebo-controlled study	IBS	31	Self-reported diarrhea, abdominal distension, abdominal pain, constipation, rectal sensitivity	Osteopathy is beneficial for improving short-term and long-term abdominal distension and pain and decreasing rectal sensitivity	3
Asthma	Hondras et al., 2005 [66]	Systematic review	Asthma	156 (pooled)	Vital capacity, FEV1, FEV1/FVC ratio, hospital admissions, hospitalization days, emergency room visits, medication use, quality of life, and subjective symptoms	Insufficient evidence to support or refute the use of manual therapy for patients with asthma	2
Cranial malformation and plagiocephaly	Pastor-Pons et al., 2021 [20]	RCT	Cranial malformation and PP	34	AROM, neuromotor development, ODDI	PIMT is effective for cervical movement limitation in infants with PP	3
	Billi et al., 2017 [44]	Pilot clinical standardization project (pre-post design)	Cranial malformation and NSOP	10	ODDI, EDI, CPI	Functional MT may be beneficial for NSOP, further research needed	2
	Lessard et al., 2011 [45]	Pilot clinical standardization project (pre-post design)	Cranial malformation and NSOP	12 + 5 (initial moderate to severe CVA)	CVA, SBA, TCVA	Osteopathic treatments may contribute to improving cranial asymmetries in infants with NSOP, further RCT needed	3
Torticollis	Pastor-Pons et al., 2021 [20]	RCT	Torticollis and PP	34	AROM, AIMS, ODDI	PIMT is effective for cervical movement limitation in infants with PP	3
	Ellwood et al., 2020 [46]	Systematic review	PP, CMT	14 systematic reviews	Change in head shape irrespective of outcome measures used (for PP and CMT)	MT is effective for treating PP, practitioner-led stretching is moderately effective for increasing range of movement in CMT	2
Scoliosis	Théroux et al., 2017 [49]	Systematic review	IS	N/A (4 studies)	Primary: Cobb angle. Secondary: aesthetics, pain intensity, physical disability, quality of life, and adverse events.	Insufficient evidence to establish whether spinal MT may be beneficial for adolescent IS	3
	Płaszewski et al., 2014 [48]	Overview of systematic reviews	IS	(21 systematic reviews)	WHOQOL-BREF questionnaire	Insufficient evidence to determine the effectiveness of non-surgical interventions in adolescent IS	3
	Romano et al., 2008 [50]	Systematic review	IS	3	Cobb degrees	Cannot draw any conclusions on the efficacy of MT as an effective technique for the treatment of adolescent IS due to lack of serious scientific data	3
Infantile colic	Hayden et al., 2006 [52]	Preliminary open, controlled, prospective study	Infantile colic	28	Crying time, sleeping time, parental attention	Cranial osteopathic treatment can be beneficial, but a larger, double-blind study is warranted	3
	Ellwood et al., 2020 [54]	Systematic review	Infantile colic	32 studies	Crying time, sleep distress, adverse events	Probiotics strongest evidence for breastfed infants, weaker but favorable evidence for MT	2
	Dobson et al., 2012 [53]	Cochrane systematic review	Infantile colic	6 studies, 325 infants	Change in hours crying time per day. Presence/absence of colic after treatment or at later follow-up, or both, that is, the number of infants in which excessive crying resolved, Any reported adverse outcomes	Inconclusive due to small and methodologically prone to bias studies	3
Attention deficit disorder	Accorsi et al., 2014 [51]	RCT	ADHD	28	Biancardi-Stroppa Test accuracy and rapidity	OMT may potentially increase performances of selective and sustained attention in children with ADHD	3
Asthma	Jones et al., 2021 [55]	RCT	Asthma	58	Pulmonary function testing	Benefits of OMT on short-term spirometry results remain unclear, insufficient data to establish effectiveness	1
Prematurity	Lanaro et al., 2017 [47]	Systematic review and meta-analysis	Prematurity	5 RCTs, 1306 infants	Length of stay, costs	Osteopathy is beneficial, reducing length of stay and costs in preterm infants	2
Brestfeeding	Danielo Jouhier et al., 2021 [56]	Double-blind RCT	Breastfeeding	128	Exclusive breastfeeding at 1 month	OMT did not improve exclusive breastfeeding at 1 month	1
Fertility	Ruffini et al., 2016 [57]	Systematic review	Fertility	24 studies, 1840 participants	Various, including hormone levels, conception and pregnancy rate, quality of life	OMT effective for pregnancy-related back pain, uncertain for other gynecological and obstetrical conditions	2
Endometriosis	Daraï et al., 2017 [59]	Prospective cohort study	Endometriosis	46	SF-36 questionnaire, gynecological, digestive, and general symptoms	Osteopathic treatment can improve symptoms and quality of life in patients with colorectal endometriosis	2
	Daraï et al., 2015 [58]	Prospective pilot study	Endometriosis	20	SF-36 QOL questionnaire	OMT can improve QOL of patients	3
LBP and pelvic pain in pregnancy	Franke et al., 2017 [60]	Systematic review and meta-analysis	LBP and pelvic pain	5 studies for pregnancy, 3 for postpartum	Pain and functional status	Osteopathy is beneficial for pregnant or postpartum women with LBP	3
	Licciardone et al., 2010 [61]	RCT	LBP and pelvic pain	144	Back-specific functioning, back pain	Osteopathy slows or halts the deterioration of back-specific functioning during the third trimester of pregnancy	1
	Hall et al., 2016 [62]	Systematic review and meta-analysis	LBP and pelvic pain	10 studies, 1198 pregnant women	Pain intensity, acceptability of manual therapy	Limited evidence to support the use of complementary manual therapies for managing low back and pelvic pain during pregnancy	1

Summary of the recommendations for osteopathy in relation to HAS and the results in the literature (Table 4)

**Table 4:** Osteopathy indications and recommendations HAS- Haute Autorité de Santé (French National Authority for Health), N-O - no opinion, GERD - gastroesophageal reflux disease, TMJ- temporomandibular joint, ADHD - attention deficit hyperactivity disorder

	HAS recommendation	Overall scientific literature recommendation
Indication		
Preventive treatment	N-O	No high-evidence study
Common low back pain	Recommended	Recommended
Neck pain	Recommended	Non-conclusive
Idiopathic scoliosis	N-O	No high-evidence study
Sciatica/ radiculopathy	Recommended	Recommended
Cervical radiculopathy	N-O	Recommended
Arnold's neuralgia	N-O	No high-evidence study
Intercostal neuralgia	N-O	No high-evidence study
Ankle sprain	N-O	Recommended
Knee sprain	N-O	No high-evidence study
Wrist sprain	N-O	No high-evidence study
Carpal tunnel syndrome	N-O	No high-evidence study
Epicondylitis	N-O	No high-evidence study
Epitrochleitis	N-O	No high-evidence study
TMJ dysfunction	N-O	Recommended
Headaches	Recommended	Recommended
Migraines	N-O	Not recommended
Vertigo	N-O	No high-evidence study
Sleep disorders - insomnia	N-O	No high-evidence study
ADHD	N-O	No high-evidence study
Stress or burnout syndrome	N-O	No high-evidence study
GERD	N-O	Non-conclusive
Bowel transit disorders (diarrhea, constipation)	N-O	No high-evidence study
Irritable bowel syndrome	Non-conclusive	Non-conclusive
Asthma	N-O	Not recommended
Fertility issues	N-O	Not recommended
Endometriosis	Recommended	Recommended
Low back and pelvic pain in pregnant women	N-O	Recommended
Breech presentation	N-O	No high-evidence study
Nausea and vomiting in pregnant women	N-O	No high-evidence study
Plagiocephaly	Non-conclusive	Non-conclusive
Torticollis	Not recommended	Non-conclusive
Idiopathic scoliosis in children	N-O	Not recommended
GERD in children	N-O	No high-evidence study
Colic	N-O	Non-conclusive
Functional abdominal pain in children	N-O	No high-evidence study
ADHD in children	N-O	Not recommended
Premature infants	N-O	Recommended
Breastfeeding	N-O	Not recommended
Asthma in children	N-O	Not recommended
Idiopathic scoliosis	N-O	Non-conclusive
Recommendation		
No opinion/ no high-evidence study	33, 80.49%	18, 43.9%
Recommended	5, 12.2%	9, 21.95%
Non-conclusive	2, 4.88%	7, 17.07%
Not recommended	1, 2.44%	7, 17.07%

Most of the 41 indications examined (33/41, 80.49%) are not mentioned in HAS recommendations, while 18 (43.9%) lack high-evidence studies in the overall scientific literature. A total of five indications (12.2%) were recommended by HAS, and nine (21.95%) were supported by high-level evidence in the literature. The results are inconclusive for two indications (4.88%) for HAS and for seven indications (17.07%) in the literature.

Discussion

The main finding of this review is that only nine of the osteopathy indications proposed by the French osteopathy societies are justified in the literature, while five are supported by French HAS recommendations.

Our review shows that the main indication for osteopathy is the musculoskeletal system, with the greatest number of RCTs and SRs. This confirms previous reviews on this topic [2, 16]. Although it is sometimes underestimated by orthopedic surgeons, rheumatologists, and general practitioners, osteopathy has been shown to be effective for pain control and the management of certain conditions such as LBP in adults and pregnant women, acute sciatica, mild to moderate ankle sprain, and TMD [1, 6-8, 10, 19, 30, 31, 34-36, 39]. All included SRs and meta-analyses agree on the beneficial effect of OMT for nonspecific LBP [1, 10, 31], in particular, the RCT by Nguyen et al., which reported improved functional scores [30]. However, the only difference between OMT and sham groups in that study was -3.4 at three months and -4.3 at 12 months on the Quebec Back Pain Disability Index (QBPDI) scores [30], which raises concerns that results may not reach the minimally clinically important difference (MCID). Osteopathy also seems to be beneficial for acute sciatica and cervical radiculopathy [19, 34]. The benefits of osteopathy for various spinal conditions are highly important to orthopedic surgeons, who could include this technique as an adjunctive strategy for optimal results, to reduce the side effects of medication, and to limit the indication for surgery. The use of osteopathy could be even more critical for LBP and pelvic pain in pregnant women because of the limited options in this population [60]. However, it must be noted that the level of evidence for these recommendations is low. 

Moderate-level evidence was found for osteopathy in the treatment of acute ankle sprains [35, 36]. Most of the articles in the SR by Seah and Mani-Babu used OMT as an adjunctive therapy. However, it is important to note that the exact contribution of osteopathic manipulation to the observed improvements remains unclear because it is often combined with other functional treatments [36]. Although certain studies have shown that osteopathy helps improve TMD, with better mouth opening and reduced pain [6, 7, 9], the quality of evidence varies across studies, and does not clearly show that osteopathy is better than standard treatment options [6-9, 39].

The results in the literature on osteopathic treatment for neck pain are mixed, with methodological quality and levels of evidence ranging from very low to moderate, making it difficult to determine the role of this technique [18, 32, 33]. HAS recommendations are cautious for this indication [68] and associated with precautions for cervical manipulation [75]. HAS notes that cervical imaging is not always necessary in the absence of red flags. In cases of non-traumatic neck pain, imaging is immediately indicated when associated with red flags such as neurological signs and symptoms, and should be considered for episodes that last more than 4 to six weeks. Further high-quality research is needed to confirm the efficacy and safety of osteopathic treatment and improve clinical practices for neck pain management [75]. The indications for lateral and medial epicondylitis are inconclusive and report only short-term benefits, with no long-term studies [37, 38]. Osteopathy has not been shown to be beneficial for idiopathic scoliosis [5].

Headaches, in particular tension-type headaches, are a common public health problem that can significantly influence a person's quality of life [40, 41]. Our review shows that osteopathic treatment can effectively reduce the frequency and severity of headaches while improving quality of life [40, 41]. The mechanism of action of these benefits may be the reduction of pro-inflammatory substances, increase in parasympathetic tone, and the management of underlying factors that contribute to the development of headaches, such as muscle tension, joint dysfunction, and postural imbalances [40, 41, 76].

Studies show that osteopathy may reduce the number of migraines but not improve their severity [42]. However, the evidence remains inconclusive due to methodological issues and biases. The evidence for vertigo and balance disorders is also insufficient to draw clear conclusions on the effectiveness of osteopathy [43].

Our review shows that osteopathy helps reduce the length of hospital stay and the cost of treatment in premature infants [47]. The mechanisms of action include reducing pro-inflammatory substances and enhancing the lymphatic and immune systems [61, 77-79]. Other indications such as cranial malformation and plagiocephaly, torticollis, idiopathic scoliosis, infantile colic, attention-deficit/hyperactivity disorder, and asthma have mixed results and lack consistent evidence for the effectiveness of OMT [20, 44-46, 49-53, 55]. While some studies show positive outcomes for these conditions, others have methodological limitations or do not support the use of OMT.

Our review suggests that the results of osteopathy are promising in women with endometriosis [58, 59]; however, the evidence supporting this assertion is limited. The HAS recommendation also supports this potential benefit, but more robust and well-designed studies are needed [71].

The results of the included studies showed a potential benefit for the symptoms of GERD and IBS; however, the overall quality of the evidence was not strong enough to make a clear recommendation [17, 63, 65].

Although OMT is generally well tolerated [80], only a few studies included in our review clearly defined adverse effects [1, 10, 31, 60]. Among the nine indications supported by the literature, no severe adverse effects have been reported when adverse events were documented [1, 10, 31, 60, 80]. However, this cannot be confirmed for all indications. Authors must report in detail the approach used to measure adverse events, which need to be specifically defined and categorized.

Limitations

This study has certain limitations. Although some of the conclusions are based on high-level evidence, the results should be interpreted with caution due to the methodological quality of the reviews and trials, which were sometimes low. Blinding was an issue in most of the RCTs due to the difficulty of blinding patients to the osteopath and the absence of sham therapy in the control group. There may also be a performance bias because patients in an RCT receive special care from the osteopath, which could affect the outcomes. The difference in the quality of the included studies is also a limitation. The methodology of the systematic review involved the exclusion of lower-level evidence studies when higher-level evidence was available; this approach may have resulted in the exclusion of relevant studies that could have contributed to a comprehensive analysis. Thus, the results should be extrapolated with caution. The types and duration of the osteopathic treatment also varied, and most studies focused on the short-term outcomes of therapy.

Despite these limitations, these results have important implications for both clinical practice and future research. Physicians should consider incorporating osteopathic therapy into their treatment plans.

## Conclusions

In conclusion, this systematic review shows that osteopathy has evidence-based benefits for numerous conditions, in particular for musculoskeletal and neurosensory indications. However, more high-quality studies are needed to evaluate and validate the effectiveness of this technique for other indications. Recommendations from the French HAS and in the literature provide a valuable basis for clinicians and patients to understand the potential applications of osteopathy in healthcare. HAS does not recommend systematic radiography of the cervical spine before manipulation.

## Appendices

Table 5 present the French representing orgranization of osteopaths depending on their profile.

**Table 5:** French representating orgranization of osteopaths depending on their profile

Osteopath profile	Organization
Osteopaths who are not healthcare professionals	OF (Ostéopathes de France) and SFDO (Société Française de l'Ostéopathie)
Osteopaths who are physiotherapists	FFMKR (Fédération Française des Massages Kinésithérapeutes Rééducateurs) and SNMKR (Syndicat National des Masseurs Kinésithérapeutes Rééducateurs)
Osteopaths who are physicians	ODF (Ostéos de France) and SMMOF (Syndicat de la Médecine Manuelle et Ostéopathique de France)

Table 6 represents the indications for osteopathy as proposed by representative organizations.

**Table 6:** Indications for Osteopathy as Proposed by Representative Organizations FFMK - Fédération Française des Masseurs-Kinésithérapeutes pour les Techniques Ostéopathiques, AMKO - Association Médicale pour la Kinésithérapie Ostéopathique, SNMKR - Syndicat National des Masseurs-Kinésithérapeutes Rééducateurs, ENT- Ear Nose Throat

Organization	Indications & Remarks
Syndicat de Médecine-Manuelle Ostéopathie de France (Médecins-ostéopathes)	Treats reversible functional disorders. Excludes serious illnesses, permanent deformities, congenital malformations, infectious diseases. Techniques primarily for spine, limbs (joint, muscular, tendon issues). Less emphasis on cranial or visceral treatments.
Ostéos de France (Médecins-ostéopathes)	Addresses functional disorders, focusing on pain or disruptions in the musculoskeletal system and impact on certain internal organs. Excludes conditions requiring surgery, severe tissue damage, and infections.
FFMK & AMKO	Treats functional disorders of the human body, excluding organic pathologies needing medical, surgical, pharmacological intervention. Techniques are musculoskeletal and myofascial, manual and external. Referral to a physician is required for symptoms outside the practitioner's competence.
SNMKR et Union des Masseurs Kinésithérapeutes Ostéopathes	No formalized indications
Syndicat Français des Ostéopathes (Non-professionnels de santé)	Indications include lower back pain, back pain, neck pain, some forms of sprains, some tendinopathies, sports injuries, dental occlusion disturbances, headaches, migraines, certain forms of dizziness, state of hypernervosity, anxiety, sleep disorders, stress, fatigue, irritability, neuralgia, digestive disorders, genital-urinary disorders, sequelae of trauma including road accidents and consequences of falls and impacts, post-surgical work.
Ostéopathes de France (Non-professionnels de santé)	In pregnant or postpartum women: nausea, vomiting, digestive disorders, lower back pain, irritability, restoring mobility of pelvis and abdomen, improving flexibility of surrounding tissues, pain, gastroesophageal reflux, fatigue, carpal tunnel syndrome, postpartum issues including pain, restoring comfortable posture, perineal rehabilitation. In infants: conditions following difficult deliveries or interventions, tension, crying, sleep issues, digestive problems, asymmetry, ENT disorders. In children: sleep disturbances, ENT disorders, behavioral issues, digestive problems, spine deviations, postural disorders, concentration issues, static disorders like scoliosis, orthodontics support. Related to sleep apnea and spinal pain.
